# The effect of non-steroidal anti-inflammatory agents on behavioural changes and cytokine production following systemic inflammation: Implications for a role of COX-1

**DOI:** 10.1016/j.bbi.2009.11.006

**Published:** 2010-03

**Authors:** J.L. Teeling, C. Cunningham, T.A. Newman, V.H. Perry

**Affiliations:** a CNS Inflammation Group, School of Biological Sciences, University of Southampton, Bassett Crescent East SO16 7PX, UK; b School of Biochemistry and Immunology, Trinity College Institute of Neuroscience, Lloyd Building, Trinity College Dublin, Ireland

**Keywords:** Cytokines, Behaviour, Systemic inflammation, Indomethacin, COX-1, COX-2

## Abstract

Systemic inflammation gives rise to metabolic and behavioural changes, largely mediated by pro-inflammatory cytokines and prostaglandin production (PGE_2_) at the blood–brain barrier. Despite numerous studies, the exact biological pathways that give rise to these changes remains elusive. This study investigated the mechanisms underlying immune-to-brain communication following systemic inflammation using various anti-inflammatory agents.

Mice were pre-treated with selective cyclo-oxygenase (COX) inhibitors, thromboxane synthase inhibitors or dexamethasone, followed by intra-peritoneal injection of lipopolysaccharide (LPS). Changes in body temperature, open-field activity, and burrowing were assessed and mRNA and/or protein levels of inflammatory mediators measured in serum and brain.

LPS-induced systemic inflammation resulted in behavioural changes and increased production of IL-6, IL-1β and TNF-α, as well as PGE_2_ in serum and brain. Indomethacin and ibuprofen reversed the effect of LPS on behaviour without changing peripheral or central IL-6, IL-1β and TNF-α mRNA levels. In contrast, dexamethasone did not alter LPS-induced behavioural changes, despite complete inhibition of cytokine production. A selective COX-1 inhibitor, piroxicam, but not the selective COX-2 inhibitor, nimesulide, reversed the LPS-induced behavioural changes without affecting IL-6, IL-1β and TNF-α protein expression levels in the periphery or mRNA levels in the hippocampus.

Our results suggest that the acute LPS-induced changes in burrowing and open-field activity depend on COX-1. We further show that COX-1 is not responsible for the induction of brain IL-6, IL-1β and TNF-α synthesis or LPS-induced hypothermia. Our results may have implications for novel therapeutic strategies to treat or prevent neurological diseases with an inflammatory component.

## 1 Introduction

Humans and animals are constantly exposed to the risk of infection by bacterial and viral pathogens, and sub-clinical, low grade infections are reported to account for up to 35% of all general practitioner consultations in the UK (Fleming et al., 2002). These infections can initiate a set of immune, physiological, metabolic, and behavioural responses, characterised by fever, reduced activity, reduced appetite, impaired cognitive function, anxiety and depression (Hart, 1988), also known as sickness behaviour. These behavioural changes are believed to be largely triggered by pro-inflammatory mediators that are produced by activated immune cells (Konsman et al., 2002) or by COX-2 mediated prostaglandin (PG) production in endothelial cells (Yamagata et al., 2001). More specifically, it is believed that the pro-inflammatory cytokines IL-1β (Bluthe et al., 2000a), IL-6 (Bluthe et al., 2000b; Cartmell et al., 2000) and TNF-α (Bluthe et al., 2000a) have a pivotal role in the onset of LPS-induced behavioural symptoms. These cytokines communicate with the brain by different mechanisms (Ek et al., 1998; Konsman et al., 2000), each resulting in *de novo* expression of cytokines within CNS tissues and widespread activation of resident immune-competent cells within the brain, the microglia. Cytokines are not the sole factors responsible for behavioural changes induced by systemic inflammation. For example, indomethacin, which interferes with the cyclo-oxygenase pathway, also reduces IL-1β-induced behavioural changes in mice and rats (Crestani et al., 1991; Plata-Salaman, 1991). We previously showed that a sub-pyrogenic dose of LPS (1 μg/kg), is sufficient to induce a marked reduction in burrowing behaviour (Teeling et al., 2007). Under these conditions of low grade inflammation, we showed that indomethacin completely reversed LPS-induced behavioural changes. In this model, neutralisation of peripheral IL-6, IL-1β or TNF-α did not alter the effect of LPS, suggesting an important role for PGs, and not blood-borne cytokines, in the onset of LPS-induced behavioural changes following systemic inflammation.

Increasing evidence suggests that systemic infection and inflammation impacts on various neurological diseases with an inflammatory component, including Alzheimer’s disease (AD) and stroke (Teeling and Perry, 2009). We and others have shown that the onset and progression of neurodegenerative diseases is exacerbated by systemic infection in both animal models and humans (Cunningham et al., 2009; Holmes et al., 2009, 2003), with clear evidence of increased neuronal damage and central cytokine production (Cunningham et al., 2009, 2005). The underlying pathways by which systemic infections alter brain function under diseased conditions are not known. Epidemiological studies suggested that long term use of non-steroidal anti-inflammatory drugs (NSAIDs) has a protective effect in progression to AD, but recent large randomized clinical trials, using predominantly COX-2 selective drugs, have been largely disappointing and have not shown any improvement in memory function of AD patients (Aisen, 2002). Better understanding of the biological pathways by which systemic inflammation influences brain function in health and disease may lead to novel or improve therapeutic strategies. Therefore, the aim of the present study was to further investigate the role of PGs and cytokines in immune-to-brain communication and the induction of LPS-induced behavioural changes. We show that COX-1 inhibition is crucial for reversing the effect of LPS on burrowing and open-field activity, while modulation of cytokine or COX-2 mediated PGE_2_ production does not affect LPS-induced changes in burrowing and open-field activity.

## 2 Materials and methods

### 2.1 Mice

Adult female C57/BL6 mice (>8 weeks, Harlan, UK) were used in all experiments, and were housed in groups of 5–10 on arrival, in plastic cages with sawdust bedding, for at least a week before testing. Food and water were available ad libitum. The holding room was temperature controlled (19–23 °C) with a 12:12 h light–dark cycle (light on at 0700 h). Females were used as they can be group-housed without the risk of outbreaks of aggression, and to conform to most of our previous work. All procedures were performed under the authority of a UK Home Office License in accordance with the UK animals (Scientific Procedures) Act 1986, and after Local Ethical approval by the University of Southampton.

### 2.2 LPS treatment and administration of NSAIDs

Mice received LPS derived from *Salmonella abortus* equi (L5886, Sigma, Poole, UK) at a dose of 100 μg/kg, via intra-peritoneal injection, unless stated otherwise. This dose of LPS reduces burrowing, open-field activity, changes core body temperature and gives a reproducible cytokine response in the brain (Teeling et al., 2007). Anti-inflammatory drugs were given 30–60 min prior to LPS injection as indicated in Table 1.

### 2.3 Burrowing

Burrowing was assessed as described previously (Deacon et al., 2002, 2001; Deacon, 2006; Teeling et al., 2007). Mice received appropriate pre-treatment followed by an intra-peritoneal injection of LPS or saline. Burrowing was measured between 1 and 3 h post treatment.

### 2.4 Open field

Open-field activity in mice was assessed using a Med Associates Activity Monitor (Med Associates Inc., Vermont). The open field consisted of an aluminium base (27 × 27 cm) enclosed on four sides with 0.7-cm thick acrylic sheet, surrounded by an opaque screen. Each mouse was placed in the middle of the open field and observed for 3 min. Measurement was made of total distance travelled (cm) and the total number of rears in the observation period (Felton et al., 2005). The open-field activity was measured between 3.5 and 4 h after LPS or saline injection.

### 2.5 Body temperature

Body temperature was measured using a rectal probe (Physitemp, Thermalerte TH5) that gave a rapid stabilization of the measured temperature. The mice were pre-adapted to measurements of rectal temperature for two days prior to the intra-peritoneal challenges to minimise stress effects. Body temperature was measured 4.5 h after LPS or saline treatment.

### 2.6 RNA isolation

Mice were terminally anaesthetized and transcardially perfused with heparinised saline. Brains were rapidly removed, and a thick coronal section (2 mm) taken (at approximately −2.7 to −3.7 from Bregma). The dorsal hippocampus was then punched out from this section, rapidly frozen in liquid nitrogen and kept at −80 °C until further use. Total RNA was extracted using RNeasy mini columns (Qiagen) according to the manufacturer’s instructions. Contaminating genomic DNA was degraded during extraction by use of DNase I enzyme (Qiagen). RNA samples were stored at −80 °C until assay.

### 2.7 Quantitative PCR

All equipment and reagents were supplied by Applied Biosystems Ltd. (Warrington, UK) unless stated otherwise. cDNA was generated from total RNA by the use of Taqman Gold RT reagents. The housekeeping gene glyceraldehyde-3-phosphate dehydrogenase (GAPDH) was measured in each sample by use of a rodent GAPDH Taqman kit. Assays for IL-1β, IL-6, TNF-α, COX-2 mRNA were performed as previously described (Cunningham et al., 2005). Primers used for COX-1 measurement were as follows: forward: 5′-CCA GAA CCA GGG TGT CTG TGT-3′, reverse: 5′-GTA GCC CGT GCG AGT ACA ATC-3′, probe: FAM – CGC TTT GGC CTC GAC AAC TAC CAG TG – TAMRA. As a positive control for cytokine production, RAW 294 cells were stimulated with LPS (100 ng/ml) in vitro and RNA isolated from the cells collected 24 h later. As a positive control for COX-2, LPS (2.5 μg) was stereotaxically injected into the mouse striatum and RNA was isolated 6 h later. To compare the expression of inflammatory mediators in the different experimental groups the amount of mRNA was estimated as the ratio of GAPDH.

### 2.8 Serum cytokine measurement

Blood samples (∼500 μl) were taken by cardiac puncture in terminally anaesthetized mice and collected in microfuge tubes. Samples were spun down and serum kept at −20 °C until further use. IL-1β, IL-6 and TNF-α serum levels were assessed with a sandwich-type ELISAs using a matched antibody pair (duoset ELISA development assay, R&D) according to the manufacturer’s instructions with minor modification.

### 2.9 Serum and brain PGE_2_ measurement

Serum levels of prostaglandin E metabolites were measured according manufacture’s instruction (Cayman, USA). Brain levels of prostaglandin E2 (PGE_2_) were measured according manufactures instruction (Assay designs, USA), with minor modification. Briefly, serum samples (50 μl) were diluted 1:10 in assay buffer provided by the manufacturer. Samples and standard were derivatized by adding 150 μl of carbonate buffer followed by overnight incubation at 37 °C. Samples and standards were then analysed according to manufactures’ instructions. Brain tissue was homogenized in 100 μl PBS and mixed with 1 ml 100% ethanol. After centrifugation at 3000 rpm for 10 min at 4 °C, supernatant was transferred to an empty tube and ethanol evaporated under a stream of nitrogen. Samples were resuspended in 500 μl of assay buffer and PGE_2_ levels measured according to manufacturer’s instructions.

### 2.10 Statistical analysis

Burrowing and open-field activity were analysed by one-way analysis of variance (ANOVA) followed, if significant, by Dunnett’s post-test versus controls. Data were analysed for normality using the Kolmogorov–Smirnov test and for equal variances using the Bartlett’s test. Changes in body temperature were assessed by paired Student’s *t*-test. The intervention studies were analysed by one-way analysis of variance (ANOVA) or two-way ANOVA, followed, if significant, by Bonferroni’s post-test using Graphpad Prism software. Values were expressed as mean ± SEM. A *p*-value <0.05 was considered to indicate statistical significant difference.

## 3 Results

### 3.1 The effect of different anti-inflammatory drugs on LPS-induced sickness behaviour

**Fig. 1:**
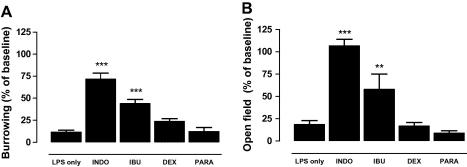
Effect of anti-inflammatory drugs on LPS-induced behavioural changes. *Burrowing:* (A) Mice were pre-treated with indomethacin (15 mg/kg), ibuprofen (15 mg/kg), dexamethasone (2 mg/kg) or paracetamol (20 mg/kg) given by intra-peritoneal (i.p.) injection. Thirty minutes later, LPS (500 μg/kg, i.p.) was administered and burrowing assayed over 2–4 h. Results were compared to baseline levels, which were obtained 24 h before the start of the experiment. Values are mean ± SEM. ∗∗∗*p* < 0.001 versus LPS alone. Data were analysed by one-way ANOVA followed by Dunnett’s test versus saline control. *Open-field activity:* (B) Mice were pre-treated with indomethacin (15 mg/kg), ibuprofen (15 mg/kg), dexamethasone (2 mg/kg) or paracetamol (20 mg/kg) given by intra-peritoneal (i.p.) injection. Thirty minutes later, LPS (500 μg/kg, i.p.) was administered and open-field activity measured between 3.5 and 4 h. Results were compared to baseline levels. Values are mean ± SEM. ∗∗∗*p* < 0.001 versus LPS alone. Data were analysed by one-way ANOVA followed by Dunnett’s test versus saline control. The total number of mice was *n* = 40 with LPS *n* = 20 and INDO, IBU, DEX and PARA each *n* = 5 per group.

We previously showed that pre-treatment of mice with indomethacin is sufficient to inhibit LPS-induced changes in burrowing activity (Teeling et al., 2007). In the present study, we aimed to further investigate these observations. We tested various well known anti-inflammatory drugs, including: indomethacin, ibuprofen, acetaminophen (paracetamol) and dexamethasone (Table 1), and measured their effect on LPS-induced changes in body temperature, burrowing and open-field activity, and production of inflammatory mediators. Mice were habituated to burrowing prior to the experiment. On the day of the experiments, mice received an intra-peritoneal injection of NSAID or saline, followed 30–60 min later by an intra-peritoneal injection of LPS or saline. Burrowing was assessed 1 and 3 h after injection of LPS, followed by measurement of open-field activity and body temperature. After the analysis of behavioural changes, mice were sacrificed and tissue collected for analysis of inflammatory mediators in serum and brain. All mice showed a similar baseline of burrowing and, as expected, systemic injection of LPS resulted in a marked suppression of burrowing (Fig. 1A, *F*_(4,39)_ = 40.99, *p* < 0.001). This behavioural change was significantly inhibited by pre-treatment with indomethacin (15 mg/kg, *p* < 0.001) and ibuprofen (30 mg/kg, *p* < 0.001), while pre-treatment with acetaminophen (20 or 100 mg/kg (data not shown)) or dexamethasone (2 mg/kg) had no effect.

**Fig. 2:**
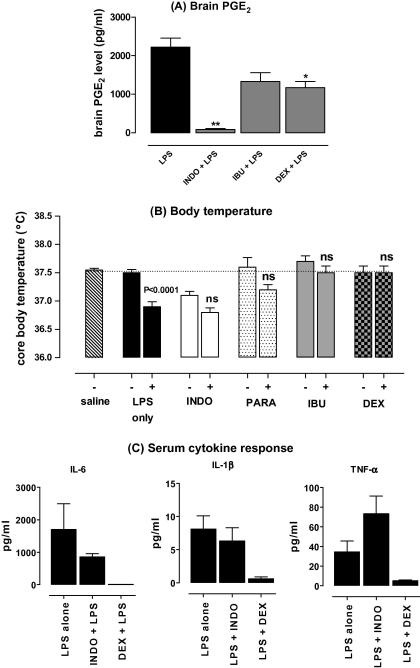
Effect of NSAIDs on PGE_2_ production and fever response to LPS. (A) Effect of indomethacin, ibuprofen and dexamethasone pre-treatment on brain PGE_2_ protein expression levels measured in punches taken through the hypothalamus taken from brains 6 h after LPS challenge. Values are mean ± SEM. Data were analysed by one-way ANOVA followed by Dunnett’s post-test. The total number of mice used in this experiment was *n* = 25 mice; *n* = 13 for LPS only, *n* = 4 for NSAIDs *p* < 0.05 is considered significantly different. (B) Effect of indomethacin, ibuprofen and dexamethasone pre-treatment on body temperature following LPS administration. Body temperature was measured using a rectal probe as described in Section 2. Baseline temperature was recorded, prior to LPS or drug injection and the results compared to saline-treated mice. Values are mean ± SEM. Data were analysed by paired Student’s *t*-test, *n* = 5 mice per group. A total of 30 mice were used in this experiment. *p* < 0.05 is considered significantly different. (C) Effect of indomethacin and dexamethasone pre-treatment on circulating cytokine production in response to LPS. Serum samples were assessed for cytokines by ELISA as described in Section 2. Values are mean ± SEM. Data were analysed by paired Student’s t-test. *p* < 0.05 is considered significantly different. *n* = 20 for LPS-only treated mice, *n* = 5 for NSAIDs. LPS was given at a dose of 500 μg/kg, after pre-treatment of mice with indomethacin (INDO, 15 mg/kg), paracetamol (PARA, 20 mg/kg), ibuprofen (IBU, 15 mg/kg), dexamethasone (DEX, 2 mg/kg), or saline as control.

The open-field activity showed a similar effect; all mice showed a similar baseline and injection of LPS resulted in a marked suppression of the number of rears (data not shown) and the total distance travelled in an open field (Fig. 1B, *F*_(4,39)_ = 23.57, *p* < 0.001). Pre-treatment with indomethacin (*p* < 0.001) and, albeit to a lesser degree, ibuprofen (*p* < 0.001) reversed the effect of LPS on open-field activity, while pre-treatment with acetaminophen or dexamethasone did not. To confirm the biological activity of the anti-inflammatory drugs used in our model, we measured PGE_2_ levels in the hypothalamus, body temperature and the circulating cytokine production. Fig. 2 shows that LPS-induced PGE_2_ levels in the hypothalamus were completely blocked by indomethacin and significantly reduced by dexamethasone (Fig. 2A, *F*_(3,24)_ = 10.92, *p* = 0.02). Although not statistically significant, ibuprofen also markedly reduced the LPS-induced PGE_2_ production in the brain. Fig. 2B shows that the LPS-induced hypothermia was completely blocked by dexamethasone and reduced by all other anti-inflammatory drugs tested. Fig. 2C shows the effect of two of the anti-inflammatory agents, indomethacin and dexamethasone, on systemic IL-6, IL-1β and TNF-α production. Indomethacin had no significant effect on LPS-induced cytokine production and even increased levels of circulating TNF-α were observed. Dexamethasone, on the other hand, completely abolished LPS-induced IL-1β, IL-6 and TNF-α production. These data suggest that, while all drugs tested were biologically active in our model, acute LPS-induced behavioural changes can only be inhibited by a subset of anti-inflammatory drugs, indomethacin and ibuprofen, and the changes in behaviour appear to be independent of blood-borne IL-6, IL-1β and TNF-α.

### 3.2 Kinetics of inflammatory mediators during systemic inflammation

**Fig. 3:**
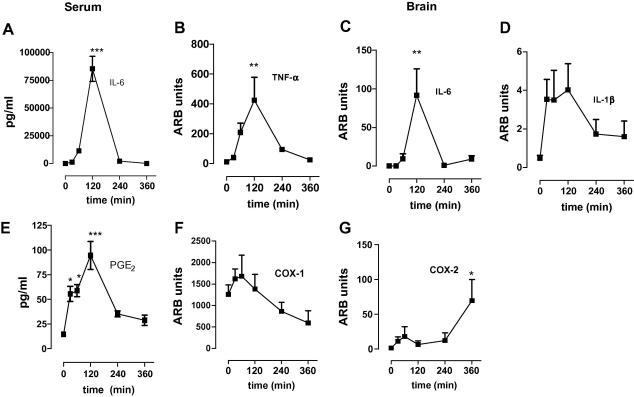
Kinetics of cytokine production in response to systemic immune challenge with LPS. (A) Effect of LPS (100 μg/kg) on expression levels of circulating IL-6 measured in serum samples taken at different time points following intra-peritoneal injection of LPS. Values are expressed as pg/ml, *n* = 5 mice per group. ∗∗∗*p* < 0.001, versus saline control (*t* = 0) with one-way ANOVA followed by Dunnett’s post-test versus saline control. (B) Effect of LPS (100 μg/kg i.p.) on relative brain mRNA expression levels of TNF-α (B), IL-6 (C) or IL-1β (D). Relative mRNA levels were measured in punches through the hippocampus taken from brain at different time points following intra-peritoneal injection of LPS. mRNA expression levels were measured by Taqman real time PCR using 40 amplification cycles. Values are relative to GAPDH expression and expressed as Arbitrary Units (ARB units). *n* = 5 mice per group; ∗*p* < 0.05 with one-way ANOVA following Dunnett’s post-test. (E) Effect of LPS (100 μg/kg) on expression levels of circulating PGE_2_ metabolites measured in serum samples taken at different time points following intra-peritoneal injection of LPS. Values are expressed as pg/ml *n* = 4 mice per group. ∗∗∗*p* < 0.001, versus saline control (*t* = 0) with one-way ANOVA followed by Dunnett’s post-test versus saline control. Effect of LPS (100 μg/kg) on relative brain mRNA expression levels of COX-1 (F) or COX-2 (G). Relative mRNA levels were measured in punches through the hippocampus taken from brain at different time points following intra-peritoneal injection of LPS. mRNA expression levels were measured by Taqman real time PCR. Values are relative to GAPDH expression and expressed as Arbitrary Units (ARB units). *n* = 5 mice per group; ∗*p* < 0.05 with one-way ANOVA following Dunnett’s post-test. A total of *n* = 30 mice was used in this experiment.

We next compared the kinetics of inflammatory mediator production in both the periphery and brain (Fig. 3). For circulating cytokines, we restricted our measurement to IL-6 since we previously showed that, in our model, this cytokine is reliably increased after LPS. Serum levels of IL-6 significantly increased at 2 h, (Fig. 3A, *F*_(1,27)_ = 47.29, *p* < 0.0001), and declined sharply to return to baseline levels by 6 h. Comparable kinetics were found for brain IL-6 production in the brain. Brain IL-6 mRNA levels increased after systemic LPS challenge (Fig. 3C, *F*_(5,24)_ = 6.381, *p* = 0.0007) showing a significant increase at 2 h and then returned to baseline by 4 h. Brain TNF-α mRNA levels increased significantly after systemic LPS challenge (Fig. 3B, *F*_(5,24)_ = 5.144, *p* = 0.0026), peaking at 2 h, after which the cytokine mRNA levels declined sharply and returned to baseline levels by 6 h. No significant changes in brain IL-1β levels were observed (Fig. 3D, *F*_(5,19)_ = 0.2683), although a trend toward increased levels was seen at 30 min.

Circulating PGE_2_ metabolite levels increased significantly after systemic LPS challenge (Fig. 3E, *F*_(1,27)_ = 14.25, *p* < 0.0001) starting at 30 min, and levels remained high for 2 h. At 6 h, PGE_2_ metabolite levels returned to baseline levels. We measured the hippocampal levels of COX-1 and COX-2 mRNA, the genes that encode the key enzymes responsible for the formation of prostanoids. All NSAIDs inhibited PGE_2_ levels in the hypothalamus (Fig. 2) and since behavioural changes were inhibited by indomethacin and ibuprofen only, we assessed the hippocampus for COX and cytokine expression levels. COX-1, changed modestly after systemic LPS challenge (Fig. 3F, *F*_(5,22)_ = 2.865, *p* = 0.0134), however, no statistically significant changes were found between *t* = 0 and any other time point after LPS. In contrast, the levels of COX-2 mRNA increased after systemic LPS challenge (Fig. 3G, *F*_(5,22)_ = 2.865, *p* = 0.0386). A small, non-significant increase was found 1 h after LPS injection and a second significant increase was observed 6 h post LPS challenge. These data suggest that PGE_2_ levels in the serum precede IL-6 production and that cytokine levels in the brain peak at 2 h.

### 3.3 The effect of specific inhibitors on LPS-induced behaviour, cytokine and prostaglandin production

**Fig. 4:**
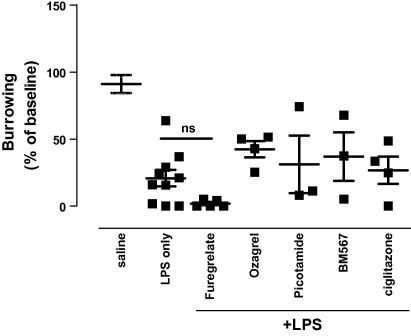
Role of thromboxane and PPAR-γ in LPS-induced behavioural changes. Mice were pre-treatment with an intra-peritoneal injection of furegrelate, picotamide, BM 567, ozagrel, ciglitazone or saline as described in Section 2, followed by a intra-peritoneal injection of LPS (100 μg/kg). Burrowing was assessed 1–3 h following LPS as described in Section 2. Values are mean ± SEM. ∗∗*p* < 0.01. One-way ANOVA followed by Dunnett’s post-test was used to analyse if behavioural changes were different from saline-treated mice. A total of 36 mice was used in this experiment: saline *n* = 8, LPS alone *n* = 8, furegrelate + LPS *n* = 5, ozagrel + LPS *n* = 4, picotamide + LPS *n* = 3, BM 567 + LPS *n* = 4, ciglitazone + LPS *n* = 4 per group.

To further investigate the biological mechanisms underlying the inhibitory effects of indomethacin and ibuprofen on LPS-induced behavioural changes, we used a series of selective inhibitors, including inhibitors of thromboxane, COX-1, COX-2 and a PPAR-γ agonist. Brain and serum samples were collected 3 h after LPS injection, immediately after the burrowing task when expression of most inflammatory mediators is still increased. Fig. 4 shows the results of pre-treatment with the thromboxane synthase inhibitors, ozagrel, picotamide, furegrelate, and the thromboxane receptor antagonist BM 567 on LPS-induced changes in burrowing. The selective inhibitors only modestly affected the LPS-induced changes in burrowing, and none of these changes were significantly different from mice treated with LPS alone (all *p* > 0.05). These data suggest that increased production of thromboxane cannot explain the effects of LPS on behavioural changes. Pre-treatment of mice with the potent and selective PPAR-γ ligand ciglitazone had no effect on LPS-induced behavioural changes (*p* > 0.05). These data suggest that direct activation of PPAR-γ does not play a role in the inhibitory effects of indomethacin on LPS-induced behavioural changes.

### 3.4 Role of COX-1 and COX-2

**Fig. 5:**
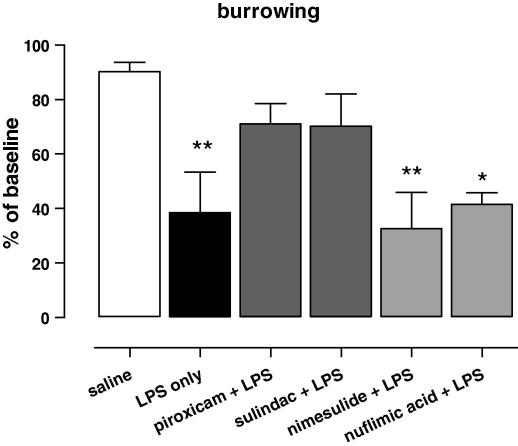
Role of COX-1 and COX-2 in LPS-induced behavioural changes. Effect of the selective COX-1 inhibitors piroxicam (10 mg/kg) and sulindac (10 mg/kg), or the selective COX-2 inhibitors nimesulide (10 mg/kg) and nuflimic acid (10 mg/kg) pre-treatment on burrowing activity measured 1–3 h following intra-peritoneal injection of LPS. Values of behaviour are expressed as percentage of baseline ± SEM. One-way ANOVA followed by Dunnett’s post-test was used to analyse if behavioural changes were different from ‘saline-treated’ mice. A total of 26 mice were used in this experiment: saline *n* = 5, LPS alone *n* = 5, piroxicam + LPS *n* = 5, sulindac + LPS *n* = 3, nimesulide + LPS *n* = 5, nuflimic acid + LPS *n* = 3 per group. ∗*p* < 0.05, ∗∗*p* < 0.01.

Thus far, our data suggest a role for COX in LPS-induced changes in burrowing and open-field activity. To investigate the role of the different isoforms of COX we next compared the effect of selective COX-1 and COX-2 inhibitors on LPS-induced behaviour changes. Fig. 5 shows the changes in burrowing tested 1–3 h after injection of LPS. Administration of LPS alone significantly decreased burrowing (Fig. 5, *F*_(5,25)_ = 4.851, *p* = 0.0046) and mice pre-treated with the COX-1 selective inhibitors piroxicam and sulindac no longer differed from saline-treated mice. In contrast, pre-treatment with the selective COX-2 inhibitor nimesulide or niflumic acid had no effect and mice were still significantly impaired in the burrowing task.

**Fig. 6:**
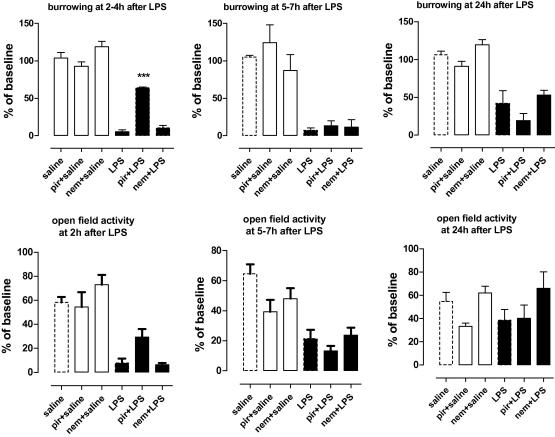
Kinetics of COX-1 and COX-2 in LPS-induced behavioural changes. Effect of the selective COX-1 inhibitor piroxicam (10 mg/kg), or the selective COX-2 inhibitor nimesulide (10 mg/kg) on burrowing and open-field activity measured 1–3, 4–6 and 24 h following intra-peritoneal injection of LPS. COX inhibitors were given 30 min prior to LPS by intra-peritoneal administration. Values are expressed as percentage of base line ± SEM, *n* = 5 mice per group. ∗∗∗*p* < 0.0001. Data were analysed by two-way ANOVA followed by Bonferroni post-test. A total of 30 mice were used in this experiment.

We next tested the effect of the inhibitors at various time points after injection of LPS to investigate the possibility that LPS-induced burrowing and open-field activity are differentially regulated over time as was previously reported for other behaviours (Swiergiel and Dunn, 2002). Fig. 6 shows the effect of LPS on burrowing and open-field activity at 2–4, 5–7 and 24 h after injection of LPS in mice pre-treated with the COX-1 specific inhibitor piroxicam or the COX-2 specific inhibitor nimesulide. The anti-inflammatory drugs were suspended in the same vehicle and given 30 min prior to LPS. Administration of LPS significantly reduced burrowing at all time points tested. Piroxicam significantly reversed the effect of LPS on burrowing when tested between 2 and 4 h (Fig. 6, *F*_(1,12)_ = 36.91, *p* < 0.0001). At later time points piroxicam was no longer protective, which may be explained by the short half life of drug in mice (*T*_1/2_ = 1.7 h) (Milne and Twomey, 1980). Nimesulide (*T*_1/2_ = 2–3 h) (Hull et al., 2005) did not significantly reverse the LPS-induced changes in burrowing at any time point tested (Fig. 6). Similar results were observed for open-field activity: a clear trend towards protection of piroxicam at 2–4 h which disappeared at later time points. Pre-treatment with the drugs alone did not have an effect on burrowing or open-field activity. Interestingly, mice pre-treated with the COX-2 inhibitor appeared to recover better 24 h after LPS injection, compared to LPS-treated only or piroxicam pre-treated mice. The changes did not, however, reach significance. These results suggest that LPS-induced changes in burrowing and open-field activity between 2 and 4 h are largely mediated by COX-1 activity and show a minimal role for COX-2.

### 3.5 Cytokines and prostaglandin production

**Fig. 7:**
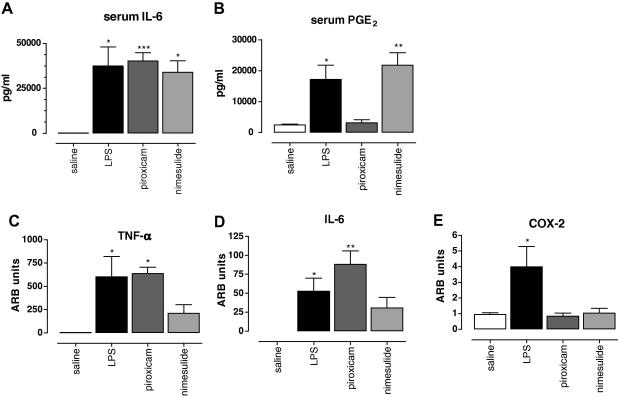
Role of COX-1 and COX-2 in LPS-induced behavioural changes and inflammatory mediator production. Effect of the selective COX-1 inhibitor piroxicam (10 mg/kg), or the selective COX-2 inhibitor nimesulide (10 mg/kg) pre-treatment on expression levels of (A) circulating IL-6 measured in serum samples taken at 3 h following intra-peritoneal injection of LPS, (B) circulating PGE_2_ metabolites measured in serum samples taken at 3 h following intra-peritoneal injection of LPS, (E) relative levels of TNF-α mRNA copies, (F) relative levels of IL-6 mRNA copies, and (G) relative levels of COX-2 mRNA copies. mRNA was measured from punches through the hippocampus taken 3 h following intra-peritoneal injection of LPS. Values of circulating inflammatory mediators are expressed as mean pg/ml ± SEM, *n* = 4–5 mice per group. ∗*p* < 0.05 one-way ANOVA followed by Dunnett’s test compared to saline. mRNA expression levels were quantified by quantitative PCR using 40 amplification cycles. Values are relative to GAPDH expression and expressed as Arbitrary Units (ARB units). *n* = 5 mice per group; * indicated statistically different as compared to saline treatment ∗*p* < 0.05 with one-way ANOVA following Dunnett’s post-test. A total of 19 mice was used in this experiment: saline *n* = 4, LPS alone *n* = 5, picotamide + LPS *n* = 5, nimesulide *n* = 5 per group.

Having established a key role of COX-1 in LPS-induced changes in burrowing and open-field activity, we next investigated the effect of piroxicam and nimesulide on cytokine and PG production. LPS increased serum IL-6 levels measured 3 h post challenge (Fig. 7A, *F*_(3,16)_ = 5.893, *p* = 0.0091). Pre-treatment with piroxicam or nimesulide did not affect the serum levels of IL-6. In contrast, circulating PGE_2_ levels, which were significantly increased 3 h after LPS (Fig. 7B, *F*_(3,17)_ = 7.885, *p* = 0.0025), were completely inhibited by pre-treatment with piroxicam (*p* < 0.05). Selective COX-2 inhibition had no effect on circulating PGE_2_ levels. Next, we measured cytokine mRNA levels in the brain. TNF-α mRNA was significantly increased 3 h after LPS challenge (Fig. 7C, *F*_(5,25)_ = 3.723, *p* = 0.0035). Pre-treatment with piroxicam did not change the mRNA levels of TNF-α in the brain, while, pre-treatment with nimesulide significantly inhibited TNF-α mRNA expression. IL-6 mRNA levels were also increased after LPS challenge (Fig. 7D, *F*_(3,17)_ = 6.263, *p* = 0.0064), and like TNF-α, only inhibited by nimesulide pre-treatment. Finally, we measured COX-2 mRNA levels, which were significantly up-regulated 3 h post LPS challenge (Fig. 7E, *F*_(3,18)_ = 4.674, *p* = 0.0017). Both piroxicam and nimesulide equally reduced COX-2 mRNA expression and were no longer different from saline-treated mice. The mechanism to explain these unexpected changes in COX-2 remain unknown, but it is possible that measurement at 3 h is too early to detect effects of the anti-inflammatory drugs tested. These data suggest that LPS-induced behavioural changes arise independent of cytokine production, and depend on COX-1 mediated peripheral and/or central PGE_2_ production. Furthermore, it suggests that cytokine synthesis in the brain, after intra-peritoneal challenge with LPS, largely depend on COX-2 signalling, and not on COX-1.

## 4 Discussion

Communication between the peripheral immune system and the brain is a well described phenomenon and underpins the metabolic and behavioural consequences of systemic infection and inflammatory diseases (Dantzer et al., 1999, 1998; Hart, 1988). Despite numerous studies, the biological mechanism(s) underlying these behavioural changes are still not fully understood. Previously, we showed a key role for PGs, and not the blood-borne cytokines IL-1β, IL-6 or TNF-α, in generating LPS-induced behavioural changes (Teeling et al., 2007). To further study the mechanisms underlying these observations, we pre-treated mice with a selection of widely-used anti-inflammatory drugs and assayed the behavioural changes and inflammatory mediator production following a systemic challenge with LPS. Pharmacological inhibition of cyclo-oxygenase enzymes COX-1 and COX-2, using indomethacin or ibuprofen, effectively attenuated the burrowing and open field response to systemic LPS-induced inflammation, while acetaminophen (paracetamol) or dexamethasone had no effect. Selective COX-1 inhibitors, piroxicam or sulindac, showed similar effects to indomethacin and ibuprofen and inhibited LPS-induced changes in burrowing and open-field activity. This effect was independent of IL-1β, IL-6 and TNF-α, generated either in the periphery or in the brain. Our findings therefore suggest a key role for COX-1, and not COX-2, in selected LPS-induced behavioural changes in normal, healthy mice.

### 4.1 The role of COX-1 in LPS-induced sickness behaviour

A systemic challenge of LPS not only results in cytokine production, but also in increased production of lipophilic molecules including prostaglandins (PGE_2_), leukotrienes, and thromboxanes, which can all contribute to behavioural changes. Apart from neutralising COX activity, it has been described that indomethacin and ibuprofen are potent inhibitors of thromboxanes (Higgs et al., 1986), while paracetamol or dexamethasone are not (Swierkosz et al., 2002). Furthermore, indomethacin and ibuprofen can directly bind and activate PPAR-γ that leads to an anti-inflammatory response that is independent of COX (Lehmann et al., 1997). The use of thromboxane inhibitors and a potent PPAR-γ agonist, however, ruled out that the LPS-induced behavioural changes in our model are mediated by these pathways and suggest a pivotal role for COX and subsequent PGE_2_ production as key players in the communication between periphery and brain. Indomethacin and ibuprofen have a much higher potency for the inhibition of COX-1 than COX-2, as demonstrated by their IC_50_ value, with indomethacin being more potent than ibuprofen (Botting, 2006; Gierse et al., 1999). We observed that indomethacin is a more potent inhibitor of LPS-induced behavioural changes and PGE_2_ production in the brain, suggesting a more important role for COX-1. In addition, nimesulide which selectively inhibits COX-2, and the steroid dexamethasone, which is known to repress transcription of NFκB-regulated genes such as cytokines and COX-2 (Adcock et al., 1999) had no effect on LPS-induced behavioural changes despite efficient blockade of peripheral IL-6, IL-1β and TNF-α production.

COX catalyses the conversion of the lipid metabolites arachidonic acid to PGs, and plays a key role in several physiological and pathological processes. The different isoforms of COX have been described as each having a distinct function in homeostasis and inflammation (Chandrasekharan et al., 2002; DeWitt and Smith, 1988). COX-1 is constitutively expressed in many cell types (Funk et al., 1991), and responsible for the production of PGs that are necessary for the regulation of physiological functions (Crofford, 1997). COX-2 is induced by diverse inflammatory stimuli (DuBois et al., 1997; Mitchell et al., 1994; O’Sullivan et al., 1992) and is responsible for the production of PGs in inflammation (Vane, 1994). It is generally believed that LPS, or cytokines produced by LPS, induce COX-2 and mPGES-1 expression in cerebral endothelial cells, with subsequent PGE_2_ production in the CNS leading to both fever and behavioural changes. (DuBois et al., 1997; Ek et al., 2001; Engblom et al., 2002; Mitchell et al., 1994; O’Sullivan et al., 1992; Yamagata et al., 2001). In this study, we show that changes in burrowing and open-field activity induced by a systemic LPS challenge are largely dependent on COX-1 activity and correlate with systemic production of PGE_2_, not cytokines.

There are a number of studies that suggest a role for COX-1 in regulating brain inflammatory responses. Firstly, transcriptional regulation of COX-2 and mPGES-1 needs at least 90 min (Cao et al., 2001; Elmquist et al., 1997), and therefore cannot explain the behavioural responses to LPS challenge observed 30 min after administration (Swiergiel and Dunn, 2002). Secondly, selective inhibition of COX-2 only partially reduces the level of PGE_2_ during acute and chronic inflammation, while indomethacin reduces PGE_2_ to undetectable levels (Langenbach et al., 1995): COX-1 may therefore contribute significantly to the local pool of PGE_2_ at the site of inflammation. Recent evidence also suggests that COX-1 and COX-2 have different functions in the brain as compared to the periphery. COX-1 is constitutively expressed in the brain, predominantly in microglia, and can be induced in endothelium during brain injury (Schwab et al., 2000). Both genetic ablation and pharmacological inhibition indicate an inflammatory role of COX-1 in the brain: this was elegantly demonstrated in COX-1 deficient mice that showed a less robust inflammatory response as compared to wild-type mice after intracerebral injection of LPS (Choi et al., 2008). Interestingly, COX-1 positive microglia are observed in various neurological diseases, including Alzheimer’s disease, Creutzfeldt Jacob disease and HIV associated with dementia, and correlate with disease severity and tissue damage (Choi et al., 2009). COX-2 is also constitutively expressed in the brain, and in particular in the hippocampus and cortical glutaminergic neurons (Choi et al., 2009). Despite the well-described direct neurotoxic effects, COX-2 has a potent anti-inflammatory function: intracerebral injection of LPS in COX-2 deficient mice results in a stronger inflammatory response and neuronal damage as compared to wild-type mice (Aid et al., 2008). It is well known that a systemic LPS challenge impacts on microglia in the healthy brain without evidence of irreversible neuronal damage (Cunningham et al., 2005; Dantzer and Kelley, 2007). Therefore, the behavioural changes observed in our model, which were already observed 30 min after injection of LPS, may be explained by activation of constitutive COX-1 expressed by microglia.

### 4.2 The role of kinetics in LPS-induced behavioural changes

COX-2 inhibitors did not significantly reverse deficits in burrowing and open-field activity tested 3, 6 or 24 h after injection of LPS, while COX-1 inhibition reversed deficits in these behavioural responses at 3 h. Both piroximide and nimesulide have a short half life in mice, but based on their IC_50_ value, a dose of 10 mg/kg is expected to be functional at 6 h after injection (Hull et al., 2005; Park et al., 2007). Our results are different from Swiergiel and Dunn who demonstrated that COX-1 plays an important role in the early changes in sickness behaviour, while COX-2 is more important at later time points, coinciding with the onset of a fever response (Swiergiel and Dunn, 2002). The latter study used a different behavioural test, i.e., sweetened milk intake, therefore, alternative explanations for the lack of effect of COX-2 specific inhibition in our study might be that different phases of behavioural changes and different types of behaviour (e.g., exploratory, anxiety, sickness) are regulated by different mediators.

### 4.3 Are different behaviours regulated by different pathways?

We show that the drugs tested in our study all reduced the hypothermic response to a systemic challenge of LPS, inhibited COX-2 expression in the hippocampus and inhibited PGE_2_ levels in the hypothalamus. Furthermore, COX-2 selective inhibitors potently inhibit LPS-induced IL-1β, IL-6 and TNF-α levels in the brain. These results are in accordance with well-accepted studies using selective pharmacological inhibitors and knockout mice that proved that the febrile response and behavioural changes induced by IL-1β, depend on COX-2 (Blatteis, 2007; Romanovsky et al., 2005; Zhang and Rivest, 2001). There are also studies showing that pharmacological cytokine inhibitors, for example dexamethasone are less effective against LPS-induced behavioural changes as compared to IL-1β-induced changes (Dunn and Swiergiel, 2000), and mPGES-1 deficient mice are not different to wild-type mice when challenged with LPS, while protected from IL-1β-induced anorexia (Pecchi et al., 2006). These studies strongly suggest that, cytokines and PGE_2_ have different effects on brain functions and/or act on different regions in the brain. Interestingly, Zhang et al. found a differential role for COX-1 and COX-2 in inducing fever and c-Fos expression, a marker for neuronal activity (Zhang et al., 2006, 2003). The COX-2 inhibitor SC-236 attenuated LPS-induced neuronal activity in specific forebrain sites including the ventromedial preoptic nucleus (VMPO) and the hypothalamic paraventricular nucleus (PVN), but not in brainstem sites such as the ventrolateral medulla (VLM), parabranchial nucleus (PB) and the nucleus of the solitary tract (NTS). The COX-1 inhibitor SC-560 showed the opposite effect, and blocked LPS-induced neuronal activity in the PVN, PB, NTS and VLM, without affecting the VMPO. The effects of systemic inflammation on brain activity are therefore not entirely dependent on COX-2 and certain responses may be regulated by COX-1. Based on these and our own results, we hypothesize that COX-2 and cytokine-mediated behaviour changes are functionally linked, while COX-1 mediated behavioural changes may occur independent of cytokines. It is worth mentioning that although dexamethasone-treated mice appeared normal and healthy, burrowing and open field were impaired after LPS challenge. These observations suggest that dexamethasone protects against classic sickness behaviours, but not behaviours associated with exploration and anxiety.

In conclusion, using a mouse model for acute systemic inflammation in otherwise healthy mice, we have shown that pharmacologic blockade of COX-1 activity results in a complete reversal of LPS-induced deficits in burrowing and open-field activity. Blockade of cytokine production, or COX-2 activity, does not alter these behavioural changes. We hypothesize that the effect of LPS in healthy, adult mice in reducing burrowing and open-field activity is largely mediated by COX-1 mediated PGE_2_ production by microglia. This study did not address the question whether COX-1 activity might have a similar protective role in LPS-induced behavioural changes in mice with an ongoing neurodegenerative disease. The scientific and commercial interest in modulating disease onset and progression in Alzheimer’s diseases using NSAIDs has been under scrutiny since clinical trials using predominantly COX-2 inhibitors, have produced disappointing results and failed to demonstrate clinical efficacy (Martin et al., 2008). A recent report compared long-term treatment of a wide range of NSAIDs and found that COX-1 inhibitors (ibuprofen, indomethacin, piroxicam) showed protective effect against the onset or progression of Alzheimer’s disease (Vlad et al., 2008). In the same study, COX-2 selective inhibitors and non-acetylated NSAIDs (salicylates) had no effect. These clinical studies emphasise the possible importance of COX-1 in neuroinflammation.

## Disclosure/conflict of interest

The authors have no financial conflict of interest.

## Figures and Tables

**Table 1:** Anti-inflammatory drugs used in this study.

	Dose (mg/kg)	Resuspended/dissolved	Supplied	Target
Indomethacin	15	0.2 M Tris–HCl, pH 8	Sigma	COX-1/2
Ibuprofen	30	Saline	Sigma	COX-1/2
Dexamethasone	2	Saline	Sigma	NFκB
Paracetamol				
(acetaminophen)	20	Saline	Sigma	COX
Ozagrel	5	Saline	Sigma	TBX synthase
Picotamide	85	10% DMSO/saline	Sigma	TBX synthase
BM 567	5	10% DMSO/saline	Cayman	TBX synthase
Furegrelate	10	Saline	Cayman	TBX receptor
Ciglitazone	10	10% DMSO/saline	Cayman	PPAR-γ
Piroxicam	10	0.2 M Tris–HCl, pH 8	Sigma	COX-1
Nimesulide	10	0.2 M Tris–HCl, pH 8	Sigma	COX-2
Niflumic acid	10	0.2 M Tris–HCl, pH 8	Sigma	COX-2
Sulindac	10	0.2 M Tris–HCl, pH 8	Sigma	COX-1

Mice were pre-treated with anti-inflammatory drugs at the dose indicated by intra-peritoneal injection, 30–60 min prior to LPS administration. A minimum of *n* = 3 per group was used.
